# The Association Between Oxygen Saturation Levels and Cognitive Decline in Patients with Cardiovascular Disorders

**DOI:** 10.1002/alz70861_108993

**Published:** 2025-12-23

**Authors:** Mohamed Khaled Ali Mohamed Ali Zid, Arwa Ayman Nagah Ahmed Shahin, Mohamed Hosni Kamel Elkellawi

**Affiliations:** ^1^ Cairo University Faculty of Medicine, Cairo, Cairo Egypt

## Abstract

**Background:**

Cognitive impairment is most commonly associated with cardiovascular disease (CVD), and chronic hypoxia, which is extremely prevalent in CVD patients, is speculated to be an etiologic factor of impairment. Reduced peripheral oxygen saturation (SpO₂) was found to enhance cognitive impairments, particularly Alzheimer's disease (AD) and vascular dementia (VD). This study seeks To determine the correlation between oxygen saturation and cognitive function of patients with cardiovascular disorder as an effort to determine if or not hypoxia is the underlying cause of cognitive impairment.

**Method:**

Cross‐sectional analysis was conducted on 120 patients of CVD in a tertiary care hospital. SpO₂ was measured through pulse oximetry and cognitive function was evaluated with the Montreal Cognitive Assessment (MoCA). The patients were divided into two groups based on SpO₂ values: normal (≥95%) and decreased (<95%). Statistical comparison was conducted to examine the relationship between MoCA and SpO₂ scores.

**Result:**

Those patients with low SpO₂ levels had reduced MoCA scores (mean 21.4 ± 3.2) compared to those patients with normal SpO₂ levels (mean 25.8 ± 2.7; *p* < 0.01). There was a moderate positive relationship between SpO₂ and MoCA scores (r = 0.49), indicating that decreased oxygen saturation is associated with increased cognitive impairment.

**Conclusion:**

The findings above show that reduced oxygen saturation can be a significant etiology of cognitive impairment in CVD patients, and this necessitates close monitoring of oxygen and immediate cognitive evaluation in CVD patients. More research is needed to explore the potential therapeutic benefit of increased oxygenation in avoiding or postponing cognitive impairment in CVD patients.

